# Bacterial Isolates and Antibiotic Resistance of *Escherichia coli* Isolated from Fresh Poultry Excreta Used for Vegetable Farming in Freetown, Sierra Leone

**DOI:** 10.3390/ijerph19095405

**Published:** 2022-04-29

**Authors:** Alie H. D. Mansaray, Dennis P. Y. Yankson, Raymonda A. B. Johnson, Francis L. Moses, Joseph Sam Kanu, Ibrahim Franklyn Kamara, Rony Zachariah, Ajay M. V. Kumar, Kalaiselvi Selvaraj

**Affiliations:** 1 Senior Agriculture Officer, Crops Division, Ministry of Agriculture and Forestry, West Wing, Youyi Building, Brookfields, Freetown 00232, Sierra Leone; dennisyankson@yahoo.com (D.P.Y.Y.); raymonda.johnson@yahoo.com (R.A.B.J.); 2 Directorate of Reproductive and Child Health, Ministry of Health and Sanitation, Youyi Building, Brookfields, Freetown 00232, Sierra Leone; franqoline@gmail.com; 3 Faculty of Basic Medical Sciences, College of Medicine and Allied Health Sciences, University of Sierra Leone, Freetown 00232, Sierra Leone; 4 National Disease Surveillance Program, Ministry of Health and Sanitation, Sierra Leone National Public Health Emergency Operations Centre, Cockerill, Wilkinson Road, Freetown 00232, Sierra Leone; samjokanu@yahoo.com; 5 Department of Community Health, Faculty of Clinical Sciences, College of Medicine and Allied Health Sciences, University of Sierra Leone, Freetown 00232, Sierra Leone; 6 World Health Organization, 21A-B Riverside, Off King Harman Road Freetown, Freetown 00232, Sierra Leone; ibrahimfkamara@outlook.com or; 7 Special Program for Research and Training in Tropical Diseases (TDR), World Health Organization, Avenue Appia 20, 1211 Geneva 27, Switzerland; zachariahr@who.int; 8 International Union against Tuberculosis and Lung Disease, 68 Boulevard Saint Michel, 75006 Paris, France; akumar@theunion.org; 9 International Union against Tuberculosis and Lung Disease, South-East Asia Office, C-6 Qutub Institutional Area, New Delhi 110016, India; 10 Yenepoya Medical College, Yenepoya (Deemed to be University), University Road, Deralakatte, Mangalore 575018, India; 11 All India Institute of Medical Sciences, Nagpur 441108, India; kalaiselvi@aiimsnagpur.edu.in

**Keywords:** one health, surveillance, antimicrobial resistance, microbial sensitivity tests, operational research, bacterial isolates, *Escherichia coli*, poultry excreta, SORT IT, Sierra Leone

## Abstract

The transfer of antibiotic resistance from animals to humans is of concern in recent times. One potential source of such transfer is the untreated poultry excreta used as manure in farming. We aim to identify bacterial isolates and antibiotic susceptibility patterns of *Escherichia coli* in poultry excreta in Sierra Leone. This was a cross-sectional study of fresh poultry excreta samples collected from four commercial poultry sites in Freetown, Sierra Leone, from June–September 2021. Bacterial isolates were tested against eight antibiotics using established standards. Of 100 samples, 93 showed *Escherichia coli* (93%): of those, eight isolates also had Salmonella (8%). *E. coli* was 100% resistant to all three ‘Watch’ drugs (erythromycin, cefoxitin and streptomycin) and tetracycline. *E. coli* was least resistant to ampicillin (12%), followed by chloramphenicol (35%). The prevalence of multidrug resistance was 95.6%. Multiple antibiotic resistance index ranged from 0.5–0.7 (optimal < 0.2), indicating high prior exposure to antibiotics in these poultries. Such high levels of resistance in *E. coli* isolated from poultry excreta could pose a serious threat to humans. We recommend (i) routine surveillance to monitor antibiotic resistance in poultry excreta, (ii) using poultry excreta as manure only after treatment and (iii) restricting the use of antibiotics as prophylactics and growth promoters in poultry feeds.

## 1. Introduction

Globally, there has been an increasing demand for livestock and poultry products to meet the needs of rapid urbanisation and commitment to achieve food security as part of the Sustainable Development Goals (SDGs) [1]. The use of antibiotics in sub-therapeutic concentrations in livestock and poultry farms has gained rapid attention since the Food and Drug Administration (FDA) approved 18 classes of antibiotics for growth promotion [2]. This has been postulated to promote the growth of antibiotic-resistant bacteria as well as the emergence of new resistant variants [3].

Globally, there were 4.95 million deaths in 2019 associated with antimicrobial resistance (AMR) in humans with the highest age-adjusted death rates in Western Sub-Saharan Africa [4]. Apart from the role of indiscriminate use of antibiotics in humans, the non-human use of antibiotics in agriculture and food producing animals also plays a major role in the occurrence of AMR. The non-human use of antibiotics is widely prevalent in countries such as India, China and South Africa [3]. Globally, the use of antibiotics among human beings is equivalent to non-human use either for growth promotion or for mass prophylaxis (humans: 118/population corrected unit; animals: 133 mg/kg livestock) [5]. The World Health Organization (WHO) reports that 57% of the antibiotics used as growth promoters are in the list of essential antibiotics for human use [6]. In the recent decade, in European countries, tetracycline, penicillin and sulfonamides are the most predominantly used antibiotics in animal foods as growth promoters [6]. Some of these antibiotics are poorly absorbed in livestock; hence, there is a risk of active residuals of these antibiotic agents to be present in excreta [3,7]. Poultries and livestock were identified to be the major hot spots for AMR [3,8].

Considering the threat of antibiotic resistance, the majority of developed countries have banned the use of antibiotics as growth promoters among food producing animals [2,9]. Following this, many studies have demonstrated that the restriction of antibiotics in animal food can have a great impact on the reduction of AMR [9]. However, in low-income countries such as Sierra Leone, these restrictions are not in place. Moreover, limited laboratory capacity and lack of surveillance mechanisms in these countries act as major challenges to monitor the impact of antibiotic use among food producing animals on AMR [10].

Poultry excreta, consisting of faeces, feathers, left-over feeds and nesting materials, is a cheap fertilizer for crop farming and contributes to improved crop yield and quality [11]. For this reason, it is widely used globally as organic manure. Its production has also increased [11], given the expansion of the poultry industry in all regions across the world. However, contamination of excreta with various pathogens such as bacteria, viruses, fungi and parasites remains a significant problem [7]. In Sierra Leone, poultry excreta remain one of the major sources of agricultural manure and it is a prevailing practice to use fresh excreta as manure without subjecting it to any treatment.

*Escherichia coli* (*E. coli*), Salmonella and Campylobacter species are common foodborne bacteria that have been isolated from poultry excreta [12,13]. There is a risk of transmission of these bacteria to humans, animals and the environment. Enteric pathogens remaining in the poultry excreta can act not only as pathogenic organisms among humans, but can also enhance the development and spread of antibiotic resistance. People who are at risk of being in direct contact with these poultry are at a higher risk of acquiring these resistant organisms [9]. The risk is further increased by the ability of the enteric pathogens from poultry excreta to survive up to seven months in the soil if the manure is not treated [14]. Studies from West and Central African countries have reported the wide prevalence of multidrug resistance bacteria in poultry excreta [15,16].

Poultry farmers in Sierra Leone often use antibiotics as prophylactics against infections and as growth promoters. Such exposure to antibiotics results in commensal bacteria such as *E. coli* developing resistance to the commonly used antibiotics in the poultry population [17]. Studies in farms and amongst farmers have revealed the presence of bacteria in the meat of poultry and other products [18]. The use of poultry excreta as manure for vegetable farms results in these bacteria coming into contact with the human population and the environment, resulting in the transmission of AMR from the poultry population to the human population through the One Health chain [8].

Resistance to antibiotics in *E. coli* is used as a sensitive indicator of circulating community bacterial resistance in animals. Moreover, there is a risk of transmission to humans if the *E. coli* that are isolated are resistant [19].

A recent systematic review regarding AMR in African countries highlighted the lack of data from economically poor African countries such as Sierra Leone, Mali and Burkino Faso [20]. Although poultries are predominant in number and poultry manure is being widely used as manure, there is no data available from Sierra Leone regarding the existence of pathogenic microorganisms and their AMR patterns in poultry excreta. We thus conducted this study with the following aim and objectives:

In samples of poultry excreta collected from four operational poultry farms in Freetown, Sierra Leone, between June and September 2021, to (i) determine the presence of bacterial isolates; (ii) describe the antibiotic resistance pattern of *E. coli* isolates based on the ‘WHO AWaRe’ and ‘critically important antibiotics’ for human use classification; and (iii) estimate the prevalence of multidrug resistance in *E. coli* isolates.

## 2. Materials and Methods

### 2.1. Study Design

This was a cross-sectional study involving primary collection of data from commercial poultries.

### 2.2. Setting

#### 2.2.1. General Setting

Sierra Leone is situated in the West African region bordered by the Atlantic Ocean, Guinea and Liberia and has a population of about eight million people [21]. The country is divided into five political regions, 16 districts, chiefdoms, sections and villages. Agriculture as an economic activity is the main source of livelihood for about 75% of the population through a subsistence farming practice [22]. Agriculture accounts for about 25% of the country’s Gross Domestic Product (GDP), whilst livestock/poultry accounts for 6% of GDP. The most common poultry species reared in Sierra Leone include ISA-BROWN from Holland and Guinea, and Commercial Black Cockerel and Day Old Black Cockerel imported from Nigeria or Guinea. According to a livestock survey by the Food and Agricultural Organization of the United Nations (FAO), there were 1,472,718 poultry birds reared in Sierra Leone [23]. The most widely used system of rearing poultry in Sierra Leone is the extensive (free range) one, while a few farmers with capital operate or use the semi-intensive and the intensive systems, hence the low economic output from poultry production across the country.

#### 2.2.2. Specific Setting

For this study, a total of four sites were chosen from the poultries located around the Western Area districts of Sierra Leone. Compared to other districts, these districts are more urbanised. However, poultry farms from where excreta samples were collected belong to the rural area. The study sites included four poultries situated in Yams farm Waterloo, Samuel Town Waterloo, Waterloo Peninsular and Tissana village Waterloo. All these four poultry farms are large farms where huge amounts of poultry manure are generated and used for vegetable production within the Freetown Municipality through the intensive poultry system. In this system, the birds are compartmentalised depending on the age of the birds, and each compartment is served by common drinkers and feeders. Few of these poultries acknowledge the use of antimicrobials in the feeds, while others do not have a documented log for it. It is a conventional practice that when some birds are sick, there is a mass administration of antibiotics for all the birds in the poultry for prophylactic purposes. There is no defined biosecurity, training or standard operating procedures available for the care takers working in the poultry.

#### 2.2.3. Sample Collection

From the birds housed in a confined environment, a fresh excreta sample without any contamination of other scavenging materials was collected randomly in a sterile plastic container. These excreta samples were transferred with proper labels to the nearest laboratory within 24 h. This laboratory was audited in 2019 by the West Africa Health Organization and it was accredited as one of the top three laboratories for animal health in Sierra Leone.

### 2.3. Study Population and Period

The study included samples of excreta collected from poultry birds in four poultry farms of Freetown mentioned above, from June to September 2021. In two major poultries, two breeding halls were sampled from each site. Samples of excreta were collected from birds aged 8 weeks to 16 weeks. All samples were immediately transferred to the laboratory for identification of bacterial isolates and antibiotic sensitivity patterns. To avoid contamination of the excreta sample with other scavenging materials such as poultry feeds, on the day of sample collection, drinkers and feeders in the confined rooms were suspended from a central connection. Using standard personal protective equipment, the poultry attendant visited the confined room where the birds were housed. During the visit, all possible fresh excreta released from the chicks and found on the floor with moisture were collected in a sterile plastic sheet and transferred to a sterile container. Thus, from each poultry site, around 15–20 excreta samples were collected over two days.

### 2.4. Identification of Bacterial Isolates

The transferred excreta samples were thoroughly homogenised in micro tubes with an electronic homogenizer and one gram of excreta was transferred through swab sticks into sterile conical flasks. There were four types of agar developed for the purpose of this study, namely: XLD (Himedia MO31F), MacConkey (Carl Roth H&E Schnellfarbekit), Columbia (Carl Roth) and Salmonella Shigella agar. To ensure an aseptic sterilised environment, all procedures were carried out in a laminar flow cabinet. All the four different types of agar were sterilised by either autoclaving or by microwaving. Agar was allowed to cool down spontaneously to just below 36° Celsius to safe guard the thermosensitive disposable Petri dishes. Once the agar solidified, the stirred faecal matter from the sterile conical flask was transferred using a swab stick to the agar plate. This was left for 24 h. If there was no colonisation after 24 h samples were put into incubator (ESCO-Isotherm Natural convention laboratory incubator) under ambient temperature of 37 °C for another 24 h. If there was colonisation, some of these colonies were streaked into MacConkey agar and further inoculated with the help of inoculation loops and allowed to incubate for 24 h. The selected colony growth was processed using gram stains and examined under the microscope. As the morphologies of gram-negative bacteria were not specific for *E. coli* alone, further confirmation was obtained by an enterotube technique, which used *E. coli* specific enzymes and compatible markers of gas production. The enterotube is a 12-compartment plastic tube with the ability to perform 15 standard biochemical testes to identify the bacterial sub species [24,25].

### 2.5. Antibiotic Sensitivity Pattern

The culture plates that demonstrated colonisation were inoculated, and the following eight antibiotic discs from seven classes of antibiotic were added with the specific concentrations: ampicillin 2 µg, chloramphenicol 10 µg, eryhthromycin 10 µg, cefoxitin 30 µg, penicillin 1.5 I.U, streptomycin 10 µg, sulfafurazole 100 µg and tetracycline 10 µg (Abtek Biologicals Ltd., Liverpool, UK). After 24 h of the disc diffusion process, the Minimum Inhibitory Concentrations (MICs) for break points were identified. These MIC break points were determined based on Fischer’s guidelines (Vienna) adapted from the European Committee on Antimicrobial Susceptibility Testing (EUCAST) [26].

### 2.6. Data Variables and Sources of Data

The principal investigator visited the laboratory and extracted the data from laboratory records by matching the details mentioned in the labels. From the laboratory reports, the status of the presence of bacterial isolates and type of bacteria isolated were extracted. The MIC distance for each of the eight tested antibiotics against *E. coli* was recoded based on the guidelines adapted from EUCAST [26]. The antibiotic susceptibility pattern reported from the laboratory was interpreted as sensitive, low sensitive, moderately sensitive and not sensitive. Finally, low sensitive and moderately sensitive were combined and categorised as ‘intermediate’ sensitivity.

### 2.7. Data Analysis and Statistics

Data on poultry-wise status related to the presence of bacterial isolates and sensitivity to different antibiotics were entered into an excel spread sheet. The prevalence of *E. coli* and other bacterial isolates were summarised as frequencies and percentages with 95% confidence intervals (*CI*). Similarly, susceptibility to various antibiotics was presented as frequencies and percentages disaggregated by poultry type. The antibiotic susceptibility pattern of *E. coli* was shown based on the ‘WHO AWaRe antibiotic classification’ and ‘WHO Critically Important antibiotics for human use’ [27,28]. If the organism was resistant to three or more classes of antibiotics, it was considered to be multidrug resistant (MDR) [9]. The prevalence of MDR was described as percentages with 95% *CI*. In resource-poor settings, where all bacterial isolates are not subjected to the entire range of antibiotics and access to genomic analysis is poor, the multiple antibiotic resistance Index (MARI) can be used as an effective tool to determine the intensity of antibiotic resistance and to predict the previous exposure to tested antibiotics. In this study, MARI was derived from a ratio of the number of isolates found to have antibiotic resistance against the total number of antibiotics tested for that isolate [15,29,30]. A MARI value of more than 0.2 indicates the risk of high exposure to antibiotics in the poultry.

## 3. Results

### 3.1. Bacterial Isolates in Poultry Excreta

A total of 100 poultry excreta samples were tested across four commercial poultry farms to identify bacterial isolates. Of the 100 samples, *E. coli* was isolated in 93 (93%, 95%CI: 86.1–97.1%). Eight samples of 8% (95%CI: 3.5% to 15.1%) had Salmonella isolates. All salmonella isolates invariably were associated with the presence of *E. coli* isolates.

### 3.2. Antibiotic Resistance Pattern

All identified *E. coli* samples were tested against 5–8 antibiotics. All isolates had resistance to at least one of the eight tested antibiotics. *E. coli* was 100% resistant to erythromycin, cefoxitin, streptomycin and tetracycline. *E. coli* was least resistant to ampicillin (12%) followed by chloramphenicol (35%) (Table 1).

### 3.3. MDR and Phenotypic Profile

The prevalence of MDR among *E. coli* isolates was 95.6% (95% CI: 89.2–98.8%). The phenotypic profile of MARI is given in Table 2. MARI ranged from 0.5 to 0.7. The most common antibiotic resistance phenotype was the one which included the entire range of antibiotics (Table 2).

### 3.4. Antibiotic Resistance Based on ‘AWaRe’ and ‘Critically Important Antibiotics for Human Use’

**Figure 1:**
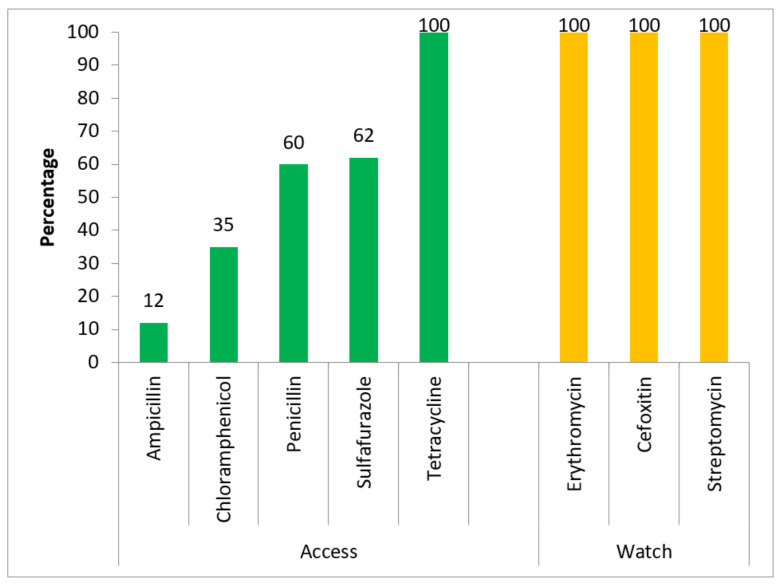
Antibiotic resistance disaggregated by the WHO AwaRe * classification for *E. coli* isolated from poultry excreta in Free Town, Sierra Leone 2021 (N = 93). * Access category: antibiotics that have activity against a wide range of commonly encountered susceptible pathogens while also showing lower resistance potential than antibiotics in the other groups. **Watch category**: This group includes antibiotics that have higher resistance potential and includes most of the highest priority agents among the “Critically Important Antimicrobials for Human Medicine” and/or antibiotics that are at relatively high risk of selection of bacterial resistance [28].

**Figure 2:**
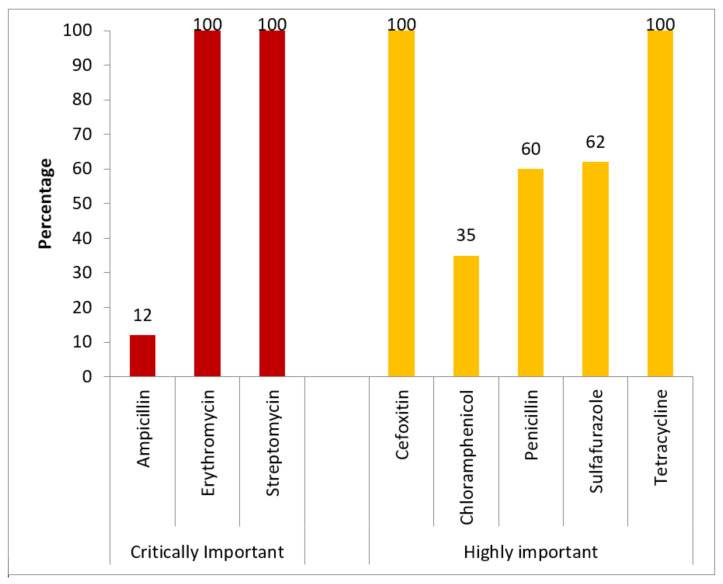
Antibiotic resistance disaggregated by WHO Critically important * classification for *E. coli* isolated from poultry excreta in Free Town, Sierra Leone 2021 (N = 93). **Critically Important**: Antibiotics fulfilling both of these following criteria: 1. Antibiotics that were the sole limited therapeutic choice to treat life-threatening bacterial infections; 2. Antibiotics used to treat infections caused by bacteria from non-human sources or with resistance genes from non-human sources. **Highly Important**: Antibiotics fulfilling only one of the following criteria: 1. Antibiotics which were the sole limited therapeutic choice to treat life-threatening bacterial infections; 2. Antibiotics used to treat infections caused by bacteria from non-human sources or with resistance genes from non-human sources [27].

All the antibiotics subjected to the antibiogram in the Watch group of antibiotics had complete resistance (100%). In the Access group of antibiotics, tetracyline had complete resistance (100%) and of the remaining antibiotics, resistance varied from 12% to 62% (Figure 1). Drugs such as streptomycin and erythromycin, which are categorised under “critically important antibiotics for human use”, showed complete resistance (100%). These drugs are reserved to treat life-threatening infections that have the potential to transmit through zoonotic origins (Figure 2).

## 4. Discussion

This is the first study from Sierra Leone reporting on bacterial isolates from fresh poultry excreta. There were three key findings. First, the prevalence of *E. coli* was very high at 93% and Salmonella was additionally isolated in 8% of samples. Second, most of the *E. coli* isolates were multidrug resistant. This is alarming given that most of the antibiotics tested were in the essential drug list for human consumption [28]. Third, *E. coli* was least resistant to ampicillin and chloramphenicol. The prevalence of *E. coli* reported in this study is in line with that reported from other settings such as the West Indies (98.5%), Senegal (84.5%) and Nigeria (90%) [16,32,33]. Some of the studies from Cameroon [15] and North West Ethiopia [34] reported a prevalence of about 45%. The isolation of *E. coli* was as low as 11% in free range birds of Nigeria in a study by Fug et al. [35]. The variability in findings may be explained by the differences in the age of the birds and the type of poultry sample used. While most studies used birds aged between 15–45 days [14,33,36], the age of the birds in the current study ranged between 8–16 weeks. Similarly, there is a wide variation in the type of poultry sample used, ranging from fresh excreta used in the current study to cloacal swabs [32,35], stored excreta up to two months [15], dried droppings from breeding halls [16], litter, water samples [36] or soil applied after poultry manure [14,36].

Susceptibility to antibiotics such as ampicillin, chloramphenicol and tetracycline are widely reported across several studies. Antibiotics such as streptomycin and erythromycin are classified as critically important antibiotics for human use, and these antibiotics are under the high prioritisation category of P3 based on their potential to transfer antibiotic resistance from *E. coli* isolates of animals to humans [27].

The level of resistance reported to erythromycin in the present study (100%) is higher compared to other studies, where the resistance varied from 40–70% [16,32,34]. Similarly, the resistance to tetracycline reported in the present study (100%) is also high compared to other studies, which varied from 40–89% [16,32,34,35,36]. The high resistance to streptomycin reported in this study (100%) is in line with other studies reported from Nigeria by Ibrahim et al. (100%), from Cote d’Ivoire by Assoumy and colleagues (72%) [37] and from Canada by Merchant et al. (80.3%) [14]. The extensive antibiotic resistance for tetracycline and erythromycin could be due to the wide use of tetracycline and macrolides in animal feeds [38].

The prevalence of ampicillin resistance in the present study was 12%. This is contrary to other studies from Africa, which reported resistance from 84–96%. However, studies which identified isolates from poultry litter and soil had reported a relatively lower prevalence (70%) and one of the similar studies by Assoumy et al. from Cote d’Ivoire also reported a low prevalence of resistance to ampicillin (49%) compared to other antibiotics [37].

Similar to ampicillin, the prevalence of chloramphenicol resistance in the present study was found to be low, at 35%. This is in contrast to chloramphenicol resistance reported by Ibrahim et al. (90%) [16] and Fug (67%) [35]. However, lower levels of resistance to chloramphenicol were reported from Nigeria (22%), North West Ethiopia (11%), the West Indies (7%) and Cote d’Ivoire (22%) [32,34,36,37]. Studies in which free range birds were included have reported lower levels of resistance, as the exposure to the antibiotics in term of feeds or mass prophylaxis could be less in these birds. The low level of resistance to chloramphenicol could be due to the non-inclusion of this antibiotic in poultry feeds, and in African counties the use of this antimicrobial is considered to be illegal.

A limited range of studies have reported antibiotic resistance to sulfa drugs. The resistance to the sulfa drug reported in the current study (62%) was lower compared with the estimates by Assoumy et al. (86%) [37]. As sulfonamides are widely available and used against parasitic coccidiosis infections, which are a source of major economic loss in the poultry, high resistance to sulfonamides is expected [39,40].

The most concerning threat was the extensive MDR reported from poultry excreta in our study and across all the studies from poultry settings. The majority of the African studies have reported an MDR prevalence of more than 80% [15,16,19,33], in contrast to a developed country such as Canada, which has reported a relatively lower MDR of 40% [14].

MARI has been used in a few studies from low- and middle-income countries as a surrogate to track the source and predict prior exposure of antibiotics to the organism. The MARI reported from these countries varied from 0.6–0.9 [15,16] as against 0.4 to 0.7 reported in this study.

In the present study, we observed a site-wise difference in the level of antibiotic resistance. Although in this study we did not have detailed characteristics of these poultries, other studies that captured the hygiene practices, knowledge of poultry attendants and antibiotic use in the poultry have identified differences between poultries [15,35].

The extensive antibiotic resistance identified in this study compared to other studies may be due to several reasons, including the following: (i) inclusion of mature birds (aged 8–16 weeks) with a higher likelihood of repeated infections, repeated exposure to antibiotics and consequent development of resistance; (ii) use of low-dose antibiotics in the poultry feeds; and (iii) methodological differences in identifying the MIC break points while interpreting the study findings. This study used EUCAST standards in contrast to the majority of other studies where Clinical and Laboratory Standards Institute CLSI guidelines have been followed.

There are some strengths of our study. First, to the best of our knowledge this is the first study from Sierra Leone which reports on antibiotic resistance patterns from poultry excreta. Second, all antibiotic sensitivity assessments were conducted in one of the accredited laboratories of Sierra Leone. As all the steps in the isolation of bacteria and antibiotic sensitivity assessments were conducted using a laminar flow, this ensured a highly sterilised laboratory environment.

We had some limitations. First, the present study findings are based on the bacterial isolates identified from commercial broiler-based poultries. In this study, we did not examine any excreta samples from free range birds, which are extensively managed as domestic operations and which contribute to more than 75% of poultry operations in the country. Hence, our findings cannot be extrapolated to free range birds.

Second, we did not perform any molecular analysis to identify the antibiotic resistant genes. To overcome this limitation, we estimated the MARI values. In resource poor countries, where molecular analysis is not feasible, MARI is being used as a simple cost effective tool to track the source or indicate prior exposure to the tested antibiotics [29,30]. In this study, the MARI was higher than 0.2, indicating prior exposure to antibiotics.

Third, in this study we did not have the data to explain the high prevalence of antibiotic resistance including MDR. Hence, whether the high AMR and MDR has occurred due to a lack of training and poor hygienic measures among poultry attendants or a high incidence of infections or the use of antibiotics in poultry feedings is not clear.

Fourth, the findings of this study came from four selected poultries of one district and hence the findings are not nationally representative. This needs future research using a nationally representative sample.

This study has the following implications. First, as the poultry excreta harbour bacterial isolates that are MDR, there is an imminent threat of the transfer of antibiotic resistance to humans when the poultry excreta are used as manure.

Second, most of the antibiotics tested in this study are often used for empirical treatment in resource poor settings. Considering the threat of transfer of AMR from poultries to humans, low-income countries that still rely on empirical treatment for curing bacterial infections must monitor the AMR of these drugs from poultry sources as well.

Third, given the high MDR levels, all poultry excreta should be treated using appropriate processes, such as composting before using the excreta as manure.

Fourth, the high MARI reported in this study indicates significant pre-exposure to antibiotics in the poultry. Hence, there is an urgent need in resource poor countries such as Sierra Leone to restrict the use of antibiotics in poultry feeds as growth promoters. Similarly, there is a need to inform veterinary policies in relation to biosecurity procedures and draft guidelines regarding how mass prophylaxis is used in poultries.

As these study findings are based on one district, the Ministry of Agriculture should invest to carry out similar assessments elsewhere to see whether our findings are replicated in other districts too. It is high time to establish representative routine poultry surveillance units to monitor antibiotic resistance trends.

Finally, future studies from Sierra Leone should attempt to identify antibiotic resistant genes in poultry excreta to predict the risk of transfer of AMR to humans.

## 5. Conclusions

Poultry excreta in Sierra Leone harbour MDR *E. coli* isolates with an imminent danger of transfer to humans if untreated excreta are used as manure for vegetable farming. The wide resistance against the antibiotics of human use can threaten the effect of empirical treatment in these settings. There is a need to monitor antibiotic resistance patterns through poultry surveillance. The high level of MARI indicates considerable pre-exposure to antibiotics. There therefore needs to be much better regulation regarding restricted use of antibiotics in poultry feeds and in mass prophylaxis.

## Figures and Tables

**Table 1:** Patterns of antibiotic sensitivity for *E. coli* isolates of poultry excreta in Sierra Leone, 2021 (N = 93).

Name of Antibiotic	No. of Isolates Tested for Sensitivity	Antibiogram *					
		Sensitive		Intermediate		Resistant	
		*n*	(%)	*n*	(%)	*n*	(%)
Ampicillin	93	0	(0)	82	(88)	11	(12)
Chloramphenicol	92	26	(28)	34	(37)	32	(35)
Erythromycin	65	0	(0)	0	(0)	65	(100)
Cefoxitin	88	0	(0)	0	(0)	88	(100)
Penicillin	88	0	(0)	35	(40)	53	(60)
Streptomycin	93	0	(0)	0	(0)	93	(100)
Sulfafurazole	93	0	(0)	35	(38)	58	(62)
Tetracycline	63	0	(0)	0	(0)	63	(100)

* Susceptible/Sensitive (Susceptible; standard dosing regimen): A microorganism is categorised as “Susceptible, standard dosing regimen”, when there is a high likelihood of therapeutic success using a standard dosing regimen of the agent. **Intermediate** (Susceptible; increased exposure): A microorganism is categorised as “Susceptible, Increased exposure *” when there is a high likelihood of therapeutic success, because exposure to the agent is increased by adjusting the dosing regimen or by its concentration at the site of infection. **Resistant**: A microorganism is categorised as “Resistant” when there is a high likelihood of therapeutic failure even when there is increased exposure [31].

**Table 2:** Phenotypic antibiotic resistance profile of *E. coli* isolates from poultries in Sierra Leone 2021.

No. of Antibiotics Tested	Phenotypic Resistance Profile	No. of Isolates	MARI
Four	AMP CHL STR SFZ	5	0.50
Six	AMP CHL CXT PEN STR SFZ	20	0.56
Seven	AMP CHL CXT PEN STR SFZ TET	3	0.43
	AMP CHL ERY CXT PEN STR SFZ	5	0.60
Eight	AMP CHL ERY CXT PEN STR SFZ TET	60	0.74

MARI: Multi Antibiotic Resistance Index; AMP: Ampicillin; CHL: Chloramphenicol; STR: Streptomycin; SFZ: Sulfafurazole; CXT: Cefoxitin, PEN: Penicillin; TET: Tetracycline; ERY: Erythromycin.

## Data Availability

Data used in this study are available under the following link: This content is licenced as CC BY 4.0. It can be downloaded from the following figshare link: https://doi.org/10.6084/m9.figshare.19130255 (accessed on 7 February 2022).
